# Neutropenia caused by hairy cell leukemia in a patient with myelofibrosis secondary to polycythemia vera: a case report

**DOI:** 10.1186/s13256-018-1663-6

**Published:** 2018-04-24

**Authors:** Andreas Hanssønn Habberstad, Hoa Thi Tuyet Tran, Ulla Randen, Signe Spetalen, Ingunn Dybedal, Geir E. Tjønnfjord, Anders Erik Astrup Dahm

**Affiliations:** 1 Department of Haematology, Akershus University Hospital, Lørenskog, Norway; 2 Department of Pathology, Akershus University Hospital, Lørenskog, Norway; 3 Department of Pathology, Oslo University Hospital, Oslo, Norway; 4 Department of Haematology, Oslo University Hospital, Oslo, Norway; 5 Institute of Clinical Medicine, University of Oslo, Oslo, Norway

**Keywords:** Polycythemia vera, Myelofibrosis, Hairy cell leukemia, Neutropenia, Myeloproliferative disorders, Oral ulcer, Case report

## Abstract

**Background:**

Polycythemia vera is a myeloproliferative disease that sometimes evolves to myelofibrosis, causing splenomegaly and neutropenia. In this case report, we describe a patient with polycythemia vera and unexplained neutropenia who later turned out to also have hairy cell leukemia.

**Case presentation:**

A middle-aged Caucasian man with polycythemia vera presented to our hospital with chronic mouth ulcers. Later he developed leukopenia and pancytopenia. Bone marrow biopsies showed fibrosis. Further morphological analyses of bone marrow and blood smears revealed probable transformation into acute myeloid leukemia. However, there were also cells indicating hairy cell leukemia. Morphological and immunohistochemical analyses later confirmed the presence of hairy cell leukemia in biopsies that had been present for 3 years. Treatment with cladribine temporarily reversed the patient’s neutropenia.

**Conclusions:**

Hairy cell leukemia may mimic development to myelofibrosis in patients with polycythemia vera.

## Background

The BCR-ABL-negative myeloproliferative neoplasms (MPN) polycythemia vera, essential thrombocythemia, and primary myelofibrosis are characterized by excessive production of terminally differentiated and functional blood cells. They clinically overlap, because primary myelofibrosis sometimes presents with thrombocythemia and may be difficult to distinguish from essential thrombocythemia, whereas both essential thrombocythemia and polycythemia vera can progress to myelofibrosis [1]. All three entities have a risk of transformation to acute myeloid leukemia (AML). Polycythemia vera, essential thrombocythemia, and primary myelofibrosis are genetically characterized by clonal mutations in *JAK2*, *CALR*, or *MPL*. The MPN diseases are thought to arise from a somatically mutated hematopoietic stem cell that gives rise to all myeloid cells, B cells, and natural killer cells [2].

Patients with polycythemia vera usually live with the disease for many years, often decades, before it may transform into AML or secondary myelofibrosis [3]. The phase when polycythemia vera is about to transform to myelofibrosis is known as spent phase polycythemia vera and is characterized by reduced erythropoiesis and splenomegaly because of extramedullary hematopoiesis. More than 10% blasts in peripheral blood is unusual and may suggest a blast transformation to AML. Postpolycythemia myelofibrosis, and certainly AML, has a poorer prognosis than polycythemia vera and may therefore be considered for allogeneic stem cell transplant [4].

Hairy cell leukemia is a mature, indolent B-cell malignancy. Patients with hairy cell leukemia are characterized by fatigue, splenomegaly, and cytopenia, most often pancytopenia, and some patients may display bone marrow fibrosis [5]. The prognosis is good, and patients usually respond well to purine analogs such as cladribine or pentostatin [6, 7]. Mutation in the *BRAF* gene leading to a Val-to-Glu substitution in position 600 of the BRAF molecule (V600E) is probably the driving mutation in hairy cell leukemia [8], and a phase II trial testing the BRAF inhibitor vemurafenib in hairy cell leukemia showed promising results, with response in 25 of 26 patients [9]. We report diagnostic and therapeutic difficulties with a patient presenting with myelofibrosis secondary to polycythemia vera, further development to AML, and hairy cell leukemia. This case report illustrates that hairy cell leukemia in a patient with polycythemia vera may be interpreted as secondary myelofibrosis, that monocytopenia could have raised the suspicion of hairy cell leukemia earlier, and that AML does not always present with CD34-positive blasts.

## Case presentation

Our patient was a white man born in 1942, married with no children and without a family history of hematologic disease. He experienced migraine, had a vasectomy in 1980, and had a duodenal ulcer in 1992 treated with triple therapy. In 2009, he underwent prostatectomy for prostate cancer. In 1995, he was diagnosed with polycythemia vera with hemoglobin (Hb) 22.2 g/dl, hematocrit 0.64, white blood cells (WBC) 10.1 × 10^9^/L, and thrombocytes 162 × 10^9^/L. The patient’s main symptom was progressively worsening headache different from his previous migraine. He was treated with phlebotomy and did not receive hydroxyurea. Splenomegaly was not present at diagnosis, but in 2001 ultrasonography revealed a moderately enlarged spleen with maximum diameter of 13 cm. From 2008, his Hb levels were normal with no further need for phlebotomy. The diagnosis of polycythemia vera was supported by a positive JAK2 V617F analysis in 2013.

In 2009, the patient developed chronic aphthous mouth ulcers and was seen by different dentists and doctors. Biopsies described “a combination of acute and chronic inflammation.” The result of polymerase chain reaction (PCR) analysis for herpes simplex virus was negative. Systemic prednisolone and nonmedical measures were applied without effect.

**Fig. 1:**
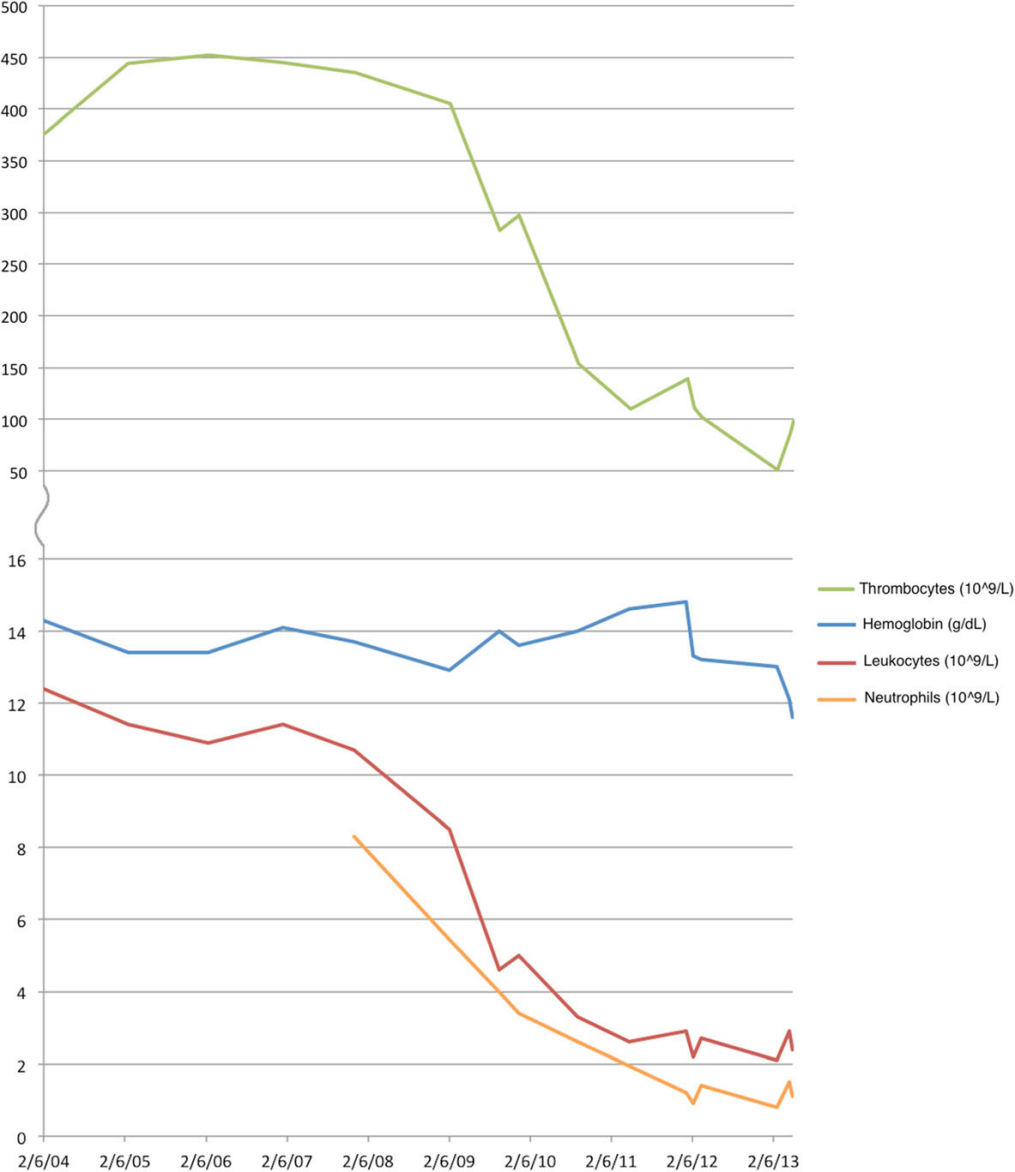
Values of hemoglobin, thrombocytes, leukocytes, and neutrophilic granulocytes during the last 9 years, showing gradual evolution of thrombocytopenia and leukopenia

The patient’s mouth ulcers persisted, and regular blood counts revealed a steady drop in neutrophils and thrombocytes with normal levels of monocytes, lymphocytes, and Hb from 2010 (Fig. 1). In March 2012, he was referred to the local department of hematology because of leukopenia and thrombocytopenia. A bone marrow biopsy showed mildly increased total cellularity (55%), increased numbers of megakaryocytes, no significant increase in erythropoiesis or granulopoiesis, normal maturing granulopoiesis, no dysplasia, no increase in CD34-positive cells, and fibrosis grade 1 interpreted as spent phase polycythemia vera. The patient’s Hb and thrombocytes decreased during the next year, and a new bone marrow biopsy in April 2013 showed increased total cellularity (60–70%), an abundance of megakaryocytes with mild dysplasia, normal erythropoiesis in numbers with some variation in size and form of nuclei, granulopoiesis with lack of normal maturation, no increase in CD34-positive cells, and fibrosis grade 2–3. Owing to excruciating mouth ulcers, filgrastim 30 million IU/week was started in May 2013. This temporarily increased the number of granulocytes, and the patient’s mouth ulcers healed after a while but later returned despite a normal granulocyte count.

In 2014, the patient again had neutropenia while receiving treatment with filgrastim. Progression of the myelofibrosis or development to AML was suspected. A bone marrow biopsy procured in April 2014 showed osteosclerosis and fibrosis grade 3, monolobulated bizarre megakaryocytes, increased erythropoiesis and myelopoiesis with reduced maturation, but no increase in CD34-positive cells. Ultrasound revealed splenomegaly with diameter 17 cm. Two additional biopsies in July and August 2014 gave similar results. Cytogenetic examination of the bone marrow from May 2014 showed a complex karyotype associated with poor prognosis with 19 different cytogenetic changes including del(7), affection of chromosome 5 and several monosomies, where del(20q) was suggested as the initial chromosomal aberration.

From May 2014, the patient was hospitalized almost monthly for fever and neutropenia. No infectional focus or microbiological agents were found. Hence, the patient received empiric antibiotic therapy with penicillin, gentamicin, piperacillin-tazobactam, ciprofloxacin, clindamycin, or meropenem. At one point, fungal infection was suspected on the basis of a positive galactomannan test result in bronchoalveolar lavage, but this result was not confirmed in blood samples. He did, however, receive treatment with voriconazole for a period. Considering progressive myelofibrosis as the main reason for the bone marrow failure, treatment with thalidomide 50 mg/day and prednisolone 30 mg/day was initiated in August 2014 [10]. This was stopped after 1 month, however, owing to a dental abscess/severe infections and no effect on blood counts.

**Fig. 2:**
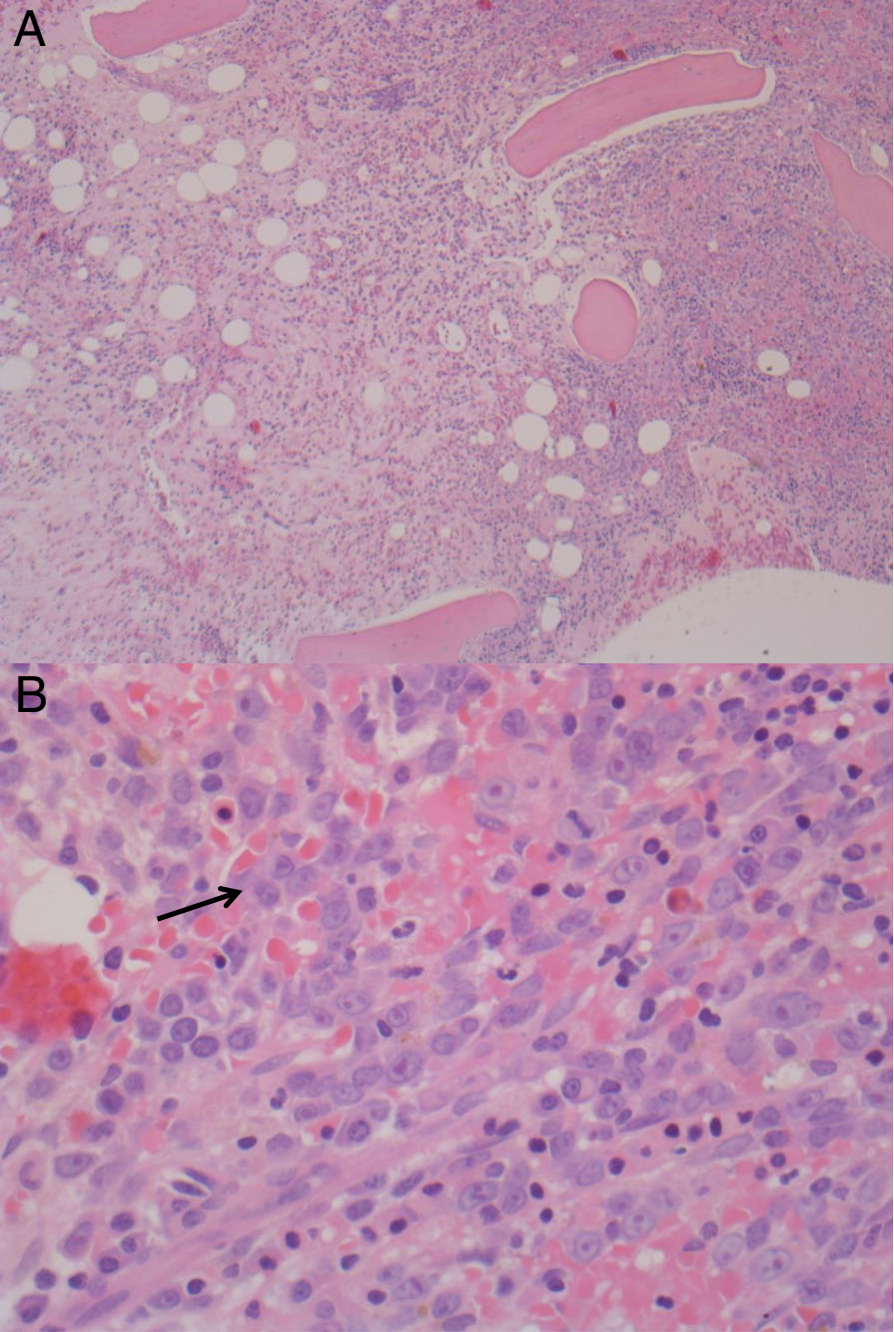
Bone marrow biopsy with hematoxylin and eosin (H&E) stain. **a** Fibrosis (left part of the picture) and increased cellularity (right part of the picture) (original magnification × 10). **b** Myeloid blasts (*arrow* at example cell) (original magnification × 60)

**Fig. 3:**
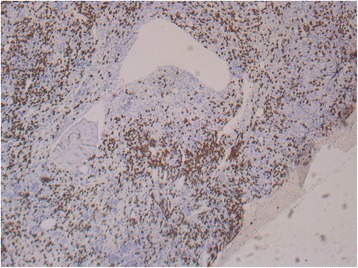
Bone marrow biopsy with immune coloring for CD20 showing hairy cells positive for CD20 in the bone marrow (brown-colored cells)

**Fig. 4:**
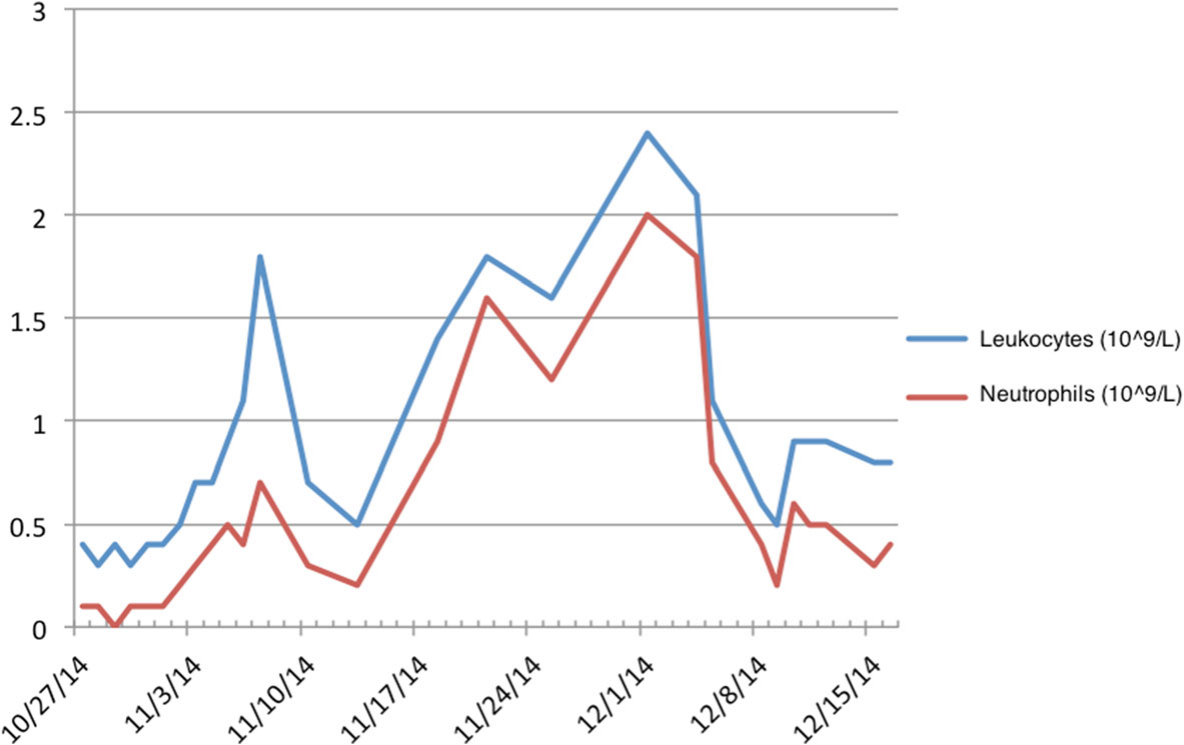
Values of leukocytes and neutrophil granulocytes after treatment with cladribine in October 2014

A new bone marrow biopsy in September 2014 disclosed immature myeloid cells with an MPO^+^, CD15^+^, CD68^+^, and CD117^+^ immunophenotype. Reexamination of previous biopsies also showed immature myeloid cells to be present in April 2014 (Fig. 2). The biopsies were sent for central review, where possible transformation to AML from 2014 was suggested. In addition, there was infiltration of CD20^+^ lymphocytes positive for cyclin D1, which constituted 15–20% of the bone marrow cells. It was concluded that this cell population was hairy cell leukemia cells (Fig. 3). In reevaluation of biopsies back to 2012, hairy cells were found in all. The patient was considered for allogeneic stem cell transplant, but owing to high risk for recurrence after transplant, it was decided to treat the hairy cell leukemia first. The patient received cladribine 0.11 mg/kg intravenously for 5 days in October 2014. This improved the number of leukocytes temporarily (Fig. 4), as well as the patient’s mouth ulcers. Shortly after this, the patient developed rash and painful edema in both legs. Biopsies confirmed perivascular infiltration of immature myeloid cells. AML then rapidly progressed with increasing bone marrow failure. The patient was given palliative care and died of pneumonia in March 2015. No autopsy was done. Table 1 shows a timeline of the case history.

**Table 1:** Timeline of case report

Date	Summaries of visits and treatments	Diagnostic testing
1995	Polycythemia vera Phlebotomy	Hb 22.2 g/dl, Hct 0.64, WBC 10.1 × 10^9^/L, TPK 162 × 10^9^/L
2008	Stable Hb Phlebotomy stopped	Hb 13.3 g/dl
2009	Mouth ulcers, prednisolone without effect	Mouth biopsy showed “acute and chronic inflammation.”
March 2012	Leukopenia, thrombocytopenia	Bone marrow biopsy showed fibrosis grade 1, “spent phase” polycythemia vera; in retrospect, also hairy cell leukemia. Hb 13.2 g/dl, WBC 2.7 × 10^9^/L, neutrophils 1.4 × 10^9^/L, monocytes 0.1 × 10^9^/L, lymphocytes 1.20.1 × 10^9^/L, TPK 102 × 10^9^/L
April–May 2013	Worsening of leukopenia, thrombocytopenia Temporary effect of filgrastim on neutrophils and partly on mouth ulcers	Bone marrow biopsy: increased cellularity and fibrosis grade 2–3; in retrospect, also hairy cell leukemia Hb 11.6 g/dl, WBC 2.4 × 10^9^/L, neutrophils 1.1 × 10^9^/L, monocytes 0.1 × 10^9^/L, lymphocytes 1.20.1 × 10^9^/L, TPK 98 × 10^9^/L
April 2014	Myelofibrosis Pancytopenia	Bone marrow biopsy with fibrosis grade 3, osteosclerosis; in retrospect, also AML and hairy cell leukemia Complex cytogenetics Hb 9.7 g/dl, WBC 2.3 × 10^9^/L, neutrophils 0.8 × 10^9^/L, monocytes < 0.1 × 10^9^/L, lymphocytes 1.40.1 × 10^9^/L, TPK 41 × 10^9^/L
May–August 2014	Repeated hospitalizations with febrile neutropenia Thalidomide and prednisolone without effect	
October 2014	Cladribine, temporary effect	Bone marrow biopsy with suggested AML and hairy cell leukemia
March 2015	Death	

Abbreviations: *Hb* Hemoglobin, *Hct* Hematocrit, *TPK* Platelet count, *AML* Acute myeloid leukemia, *WBC* White blood cells

## Discussion

The present case report is about a man with a long history of polycythemia vera who developed unexplained neutropenia, which we later found was caused by hairy cell leukemia. When we diagnosed the hairy cell leukemia, the patient had also developed secondary myelofibrosis and finally AML. The AML blasts were not CD34-positive, and it therefore took several months before we diagnosed it. We tried treatment with cladribine for the patient’s hairy cell leukemia, with short-term effect on neutropenia, but the patient later died of AML.

Patients with MPN and concomitant hairy cell leukemia have previously been reported a few times (Table 2). In a study of 267 patients with polycythemia vera followed until death, median survival was 13.5 years [11], confirming that polycythemia vera is a slowly progressing disease. In the same study, about 10% of the patients developed myelofibrosis, and 6.8% progressed to AML. Most commonly, secondary AML develops from myelofibrosis, either primary myelofibrosis or myelofibrosis secondary to essential thrombocythemia or polycythemia vera [12]. Therefore, gradual development of cytopenias and splenomegaly in a patient with polycythemia vera is an expected progression of the disease to myelofibrosis. Our patient had an additional cause of neutropenia, myelofibrosis, and splenomegaly (that is, hairy cell leukemia).

**Table 2:** Overview of previous reports of myeloproliferative neoplasms and hairy cell leukemia

Reference	MPN	HCL	Cytopenia	Other
Mufti *et al.*, 1982 [20]	Polycythemia vera 1978	1981	Neutropenia, thrombocytopenia	
Lishner *et al*., 1984 [21]	Polycythemia vera 1967, myelofibrosis 1976	1982	Thrombocytopenia, anemia,	
Lichtman *et al*., 1998 [22]	Essential thrombocythemia 1994	HCL first, 1986	Thrombocytopenia	
Azagury *et al*., 2003 [23]	Essential thrombocythemia 1995	1999, symptomatic since 2000	Neutropenia	
Kelly *et al*., 2003 [24]	Polycythemia vera 1976	2001, HCL variant	Anemia, thrombocytopenia	
Razaq *et al*., 2005 [25]	Polycythemia vera 1992	1999, HCL variant	No	Aggressive lymphoma in prostate
Rumi *et al*., 2011 [26]	Polycythemia vera			Reported in a case series
Kotchetkov *et al*., 2012 [27]	Polycythemia vera 13 years ago	HCL variant	Anemia, thrombocytopenia	BRAF-negative
Ipek *et al*., 2016 [28]	Polycythemia vera 3 years after HCL	HCL first	Anemia at HCL diagnosis	

*HCL* Hairy cell leukemia, *MPN* Myeloproliferative neoplasms

In retrospect, the patient had hairy cell leukemia from 2012 or earlier, as seen in the bone marrow biopsies. A clinical sign that could have raised suspicion was the patient’s monocytopenia, because monocytopenia is found in 90% of patients with hairy cell leukemia [5]. In contrast, patients with polycythemia usually have normal monocyte counts [13]. When our patient was first referred to our department of hematology in 2012, he had monocytes of 0.1 × 10^9^/L, but letters from his general practitioner showed that he had monocytes of 0.3 × 10^9^/L already in 2007. A different treatment strategy could have been to treat the AML first and give the patient cladribine against the hairy cell leukemia later. Nevertheless, the cytogenetic results showed that he had a very poor prognosis even with allogeneic stem cell transplant [14].

Neutropenia is a well-known cause of oral ulcers, and our patient partly responded to granulocyte colony-stimulating factor (G-CSF/filgrastim). Treatment with G-CSF has been suggested to increase the risk of AML; however, the risk is negligible compared with the prophylactic effect on neutropenic fever in patients with cancer [15].

Our patient also had oral ulcers before neutropenia was discovered. Biopsy did not reveal a cause of the ulcers, and no viral infection was detected. Iron deficiency from the phlebotomy for years might have been the cause of the patient’s ulcers [16]. The patient had ferritin 6 μg/L in 2008 and 9 μg/L in 2010. From 2012, the patient had normal iron parameters (Fe 14 μmol/L, transferrin 2.5 g/L, ferritin 44 μg/L, transferrin saturation 23%). Thus, iron deficiency does not appear to be a probable explanation for the patient’s mouth ulcers.

Hairy cell leukemia is rarely described in patients with MPN, but there are a few cases reported in the literature (see Table 2 for an overview). It appears not to be a common molecular link between myeloproliferative neoplasms and hairy cell leukemia. The *BRAF* V600E mutation was not found in a cohort of 402 patients with various myeloid neoplasms, including 90 patients with polycythemia vera [17]. It is, however, reported in an increased frequency of lymphoid malignancies in patients with myeloproliferative diseases [18, 19], suggesting a possible link between myeloproliferative diseases and the risk of lymphoid neoplasms.

## Conclusions

Our patient’s case represents a rare presentation of hairy cell leukemia as an unexpected cause of neutropenia in a patient with postpolycythemic myelofibrosis and AML who temporarily responded to cladribine. The patient’s monocytopenia could have raised the suspicion of hairy cell leukemia earlier.

## Abbreviations

AML: Acute myeloid leukemia
G-CSF: Granulocyte colony-stimulating factor
Hb: Hemoglobin
HCL: Hairy cell leukemia
MPN: Myeloproliferative neoplasms
TPK: Platelet count
WBC: White blood cells
